# Improving Medical Student Confidence in Managing Patients With Learning Disabilities and Autism Through a Simulation

**DOI:** 10.7759/cureus.96969

**Published:** 2025-11-16

**Authors:** Daniel Refaat, Caitlin Cole, Marie-Claire Healey, Ben Taylor

**Affiliations:** 1 Education Academy, Barts Health NHS Trust, London, GBR; 2 Psychiatry, East London NHS Foundation Trust, London, GBR; 3 Radiology, London North West University Healthcare NHS Trust, London, GBR; 4 Education Academy and Learning Disability, Barts Health NHS Trust, London, GBR

**Keywords:** autism spectrum disorder (asd), clinical confidence, educational innovation, experiential learning, inclusive communication, learning disabilities, lived experience, medical student training, simulation-based education, undergraduate medical curriculum

## Abstract

Background

People with learning disabilities (LD) and autism face significant health inequalities, partly due to clinicians’ limited confidence and lack of training in inclusive care. Undergraduate curricula often emphasise theory over practical skills. This project aimed to improve final-year medical students’ confidence through a combined lecture and immersive simulation using actors with lived experience.

Methods

Thirty-two students from Queen Mary University of London attended an introductory lecture, and eight participated in small-group simulation sessions. Scenarios were co-designed with actors with LD and autism, supported by communication aids and hospital passports. Debriefing involved actors, carers, and faculty. Confidence was assessed using pre- and post-intervention Likert-scale surveys, with qualitative feedback collected.

Results

Students reported improved confidence across all measured domains (p < 0.001). All simulation participants strongly agreed that the teaching should be mandatory. Qualitative feedback highlighted authenticity, safe practice opportunities, and the value of direct lived experience.

Conclusion

The innovation provided both knowledge transfer and experiential practice. Simulation offered a safe environment for communication and skill development, aligning with Kolb’s experiential learning cycle. Involving actors with lived experience met Allport’s conditions for meaningful intergroup contact, supporting attitudinal change. The intervention aligns with the GMC *Outcomes for Graduates*, the Equality Act 2010, and the National Health Service (NHS) Long-Term Plan to reduce health inequalities. Simulation with actors with lived experience improved medical students' confidence in caring for patients with LD and autism, although the sample size of students attending could limit the power of these results. The approach is replicable with adaptations and may contribute to addressing healthcare inequalities.

## Introduction

People with learning disabilities (LD) and autism experience poorer health outcomes than the general population. The Learning from Lives and Deaths (LeDeR) review highlights that those with LD die from avoidable causes more than twice as frequently, and autistic people live on average 12 years less [1]. Barriers include clinicians’ limited understanding, low confidence, and difficulty implementing reasonable adjustments [2,3]. There are thought to be more than 1.5 million people living with LD and 700,000 people living with autism in the UK, and perhaps more that are undiagnosed [4,5]. This highlights the likelihood of clinicians encountering patients with LD in clinical environments.

Undergraduate curricula often lack structured practical teaching on inclusive communication. The General Medical Council (GMC) Outcomes for Graduates require effective communication with patients, including those with additional needs [6]. The Equality Act 2010 also places a legal duty on clinicians to make reasonable adjustments [7].

Structured contact with individuals with lived experience can improve attitudes and confidence [8]. Seminal systematic reviews established the effectiveness of simulation-based medical education, findings that have since been consistently supported by later research [9-11]. Educational theory supports this approach: Kolb describes learning as a cycle of experience, reflection, conceptualisation, and experimentation [12], while Allport’s contact theory emphasises the importance of structured, equal-status, cooperative encounters between groups [13].

This study describes the design, delivery, and evaluation of a combined lecture and simulation involving actors with lived experience of LD and autism. Therefore, this study aimed to evaluate whether participation in a combined lecture and simulation-based teaching intervention improves final-year medical students’ confidence in managing patients with LD and autism. We hypothesised that students would report increased confidence following the intervention.

## Materials and methods

Context

The intervention was delivered to final-year medical students at Queen Mary University of London. It was based at Whipps Cross Hospital, Barts Health NHS Trust.

Design

The programme had two components. The first was a lecture, attended by 32 students, which introduced key clinical considerations in the care of patients with LD and autism, barriers to healthcare, and strategies for implementing reasonable adjustments. The second was a small-group simulation attended by eight students who had signed up for the session after attending the initial lecture. Two half-day sessions were run, with students participating in pairs within each scenario. Each half-day simulation session included two scenarios. This enabled them to apply lecture content to realistic, supported clinical encounters. Participant demographics and response rates are summarised in Table 1.

Actors and preparation

Actors with LD and autism were recruited through Act Up!, a local inclusive theatre company. They were paid through Education Academy funding. Preparatory visits ensured accessibility and familiarity with the environment. Scenarios were rehearsed with faculty. For each half-day simulation, there were three faculty simulation members playing different roles within the scenario, including the patient nurse, a senior doctor to offer advice, and the LD and autism practice educator. The final faculty member used the Laerdel simulation app to adjust observation parameters. The same faculty members, LD and autism practice educator and carer, attended both half-day sessions, which took place on the same day, with one in the morning and one in the afternoon.

Simulation scenarios

**Figure 1:**
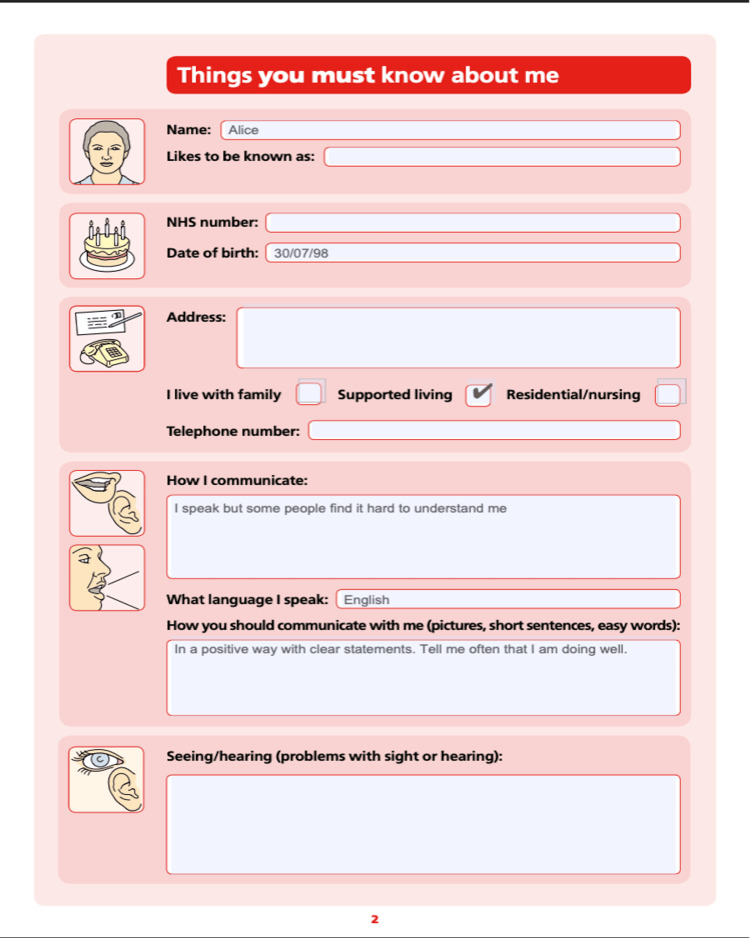
Example of a bespoke hospital passport used in the simulation The passport provided key information on communication preferences, medical history, and reasonable adjustments, adapted to each actor’s needs.

Two scenarios were developed to reflect common challenges encountered by patients with LD or autism in the hospital. The first centred on a missed injury where the patient struggled to communicate pain, while the second focused on needle phobia and the need for a capacity assessment. Scenarios were adapted to each actor’s own specific needs. Communication aids, including Makaton, visual pain scales, and hospital passports, were provided to support inclusive practice. A carer role was incorporated for authenticity. An example of the bespoke hospital passport developed for the session is shown in Figure 1.

Debriefing

Forty-minute debriefs followed each scenario that lasted for approximately 30 minutes. Each debrief was facilitated by a faculty member and an LD and autism practice educator. Actors and carers shared reflections and related the scenarios to their own experience before group discussion using a describe-analyse-apply framework.

Statistical analysis 

Pre- and post-session data were analysed according to the structure of each dataset. For the simulation component (n = 8), pre- and post-session responses were matched at the individual level; these ordinal Likert-scale data were therefore analysed using the Wilcoxon signed-rank test. For the lecture component, pre- (n = 26) and post-session (n = 32) responses could not be matched due to anonymous survey completion. As a result, these were treated as independent samples and analysed using the Mann-Whitney U test. For all items, we report mean ± standard deviation, median, and interquartile range (IQR), exact p-values, and rank-biserial effect sizes (r). All statistical tests were two-tailed with significance set at p < 0.05. Given the pilot nature of the study, statistical findings were interpreted cautiously. Quantitative data were analysed using an unpaired t-test to compare pre- and post-teaching mean confidence scores. Statistical analysis was performed using Microsoft Excel (Microsoft Corp., Redmond, WA, USA). All tests were two-tailed, and a p-value < 0.05 was considered statistically significant.

Open-text responses also captured qualitative feedback. The full pre- and post-session survey instruments used to collect both quantitative and qualitative data are provided in Appendices A-D. These include the lecture and simulation pre- and post-session questionnaires administered to students.

The full pre-/post-session survey's instrument is provided in the Appendix and was administered via Google Forms (Google LLC, Mountain View, CA, USA). Responses were collected on a six-point Likert scale (0 = strongly disagree to 5 = strongly agree).

Ethics statement

This project was conducted as an educational evaluation of a teaching intervention and was therefore exempt from formal ethics committee approval in accordance with institutional policy. Informed consent was obtained from all participating students, actors, and carers for involvement in the teaching sessions and for the use of anonymised feedback data. There is no identifiable information in the photograph of the hospital passport included in this report.

## Results

Lecture component

Across all five lecture items, post-session scores were significantly higher than pre-session scores. Mean scores increased from 1.27-2.73 pre-session to 3.69-4.00 post-session, with median scores increasing from 1-3 to 4. Mann-Whitney U tests demonstrated statistically significant improvements for all items (all p < 0.0001). Effect sizes ranged from large to extremely large (r = 0.51-0.91), indicating notable educational impact. Full results are presented in Table 2.

**Table 1:** Pre- and post-lecture knowledge scores analysed using the Mann–Whitney U test. Independent pre–post comparisons were analyzed using the Mann–Whitney U test. Effect sizes represent r (Z / √N). All comparisons were statistically significant.

Item	Mean ± SD (Pre)	Mean ± SD (Post)	Median (IQR) Pre	Median (IQR) Post	Mann–Whitney U	p-value	Effect size r
Q1. Knowledge of LD	2.54 ± 1.12	3.78 ± 0.71	3 (2)	4 (1)	629	<0.0001	0.52
Q2. Knowledge of autism	2.73 ± 0.96	3.78 ± 0.71	3 (1)	4 (1)	625	<0.0001	0.51
Q3. Knowledge of legal frameworks	1.38 ± 0.96	3.69 ± 0.90	1 (1)	4 (1)	80	<0.0001	0.83
Q4. Knowledge of reasonable adjustments	1.27 ± 0.89	3.88 ± 0.86	1 (1)	4 (1)	35	<0.0001	0.91
Q5. Knowledge of practical tools	1.73 ± 1.10	4.00 ± 0.87	2 (2)	4 (1)	98	<0.0001	0.82

Simulation component

For the simulation subgroup (n = 8), confidence improved across all three domains. Median pre-session scores ranged from 1 to 1.5 and increased to 4-4.5 post-session. Wilcoxon signed-rank tests showed statistically significant improvements for all items (p = 0.0078), with very large effect sizes (r = 0.89). These results indicate strong experiential learning benefits despite the small sample size. Full results are presented in Table 3.

**Table 2:** Pre- and post-simulation confidence scores analysed using the Wilcoxon signed-rank test. Pre–post changes analyzed using Wilcoxon signed-rank tests. Effect sizes represent rank-biserial correlation (r). All comparisons showed statistically significant improvements (p = 0.0078).

Item	Mean ± SD (Pre)	Mean ± SD (Post)	Median (IQR) Pre	Median (IQR) Post	Wilcoxon W	p-value	Effect size r
Q1. Confidence in assessing patients with learning disabilities (LD)/autism	1.50 ± 1.07	3.88 ± 0.84	1.5 (2)	4 (1)	0	0.0078	0.89
Q2. Confidence in adapting communication	1.38 ± 1.19	4.00 ± 0.93	1 (2)	4 (1)	0	0.0078	0.89
Q3. Confidence using resources	1.50 ± 1.31	4.38 ± 0.74	1.5 (2)	4.5 (1)	0	0.0078	0.89

All simulation participants strongly agreed that the teaching should be mandatory. Seventy percent of students reported that the lecture enhanced their simulation learning. All participants strongly agreed that simulation was a valuable way to learn about LD and Autism.

Qualitative analysis identified three main themes: authenticity and realism, safe and supportive learning environment, and the project's educational value and relevance. Students valued learning directly from actors, practising communication in a supportive environment, and applying resources such as hospital passports. A summary of the qualitative themes and representative student comments is presented in Table 4.

**Table 3:** Qualitative themes Student feedback is summarized under three themes: authenticity and realism (sessions felt genuine, supported by actors with lived experience of LD and autism), safe and supportive learning environment (encouraged open participation and reflection), and educational value and relevance (addressed curricular gaps and provided practical, applicable skills). Selected quotes illustrate each theme.

Theme	Description	Representative quotes
Authenticity and realism	Students valued the realism and authenticity of the session, particularly because the actors had lived experience of learning disabilities and autism. This made the learning more genuine, impactful, and reflective of real-world practice.	“Real patients.”
			“Reflection of real life.”
		“Excellent teaching. Really useful session, especially with actors who have LD & autism.”	
			“Live actors with lived experience.”
		Safe and supportive learning environment	The structure of the session fostered a safe and supportive environment, allowing students to participate openly, learn from mistakes, and engage in constructive reflection.	“Lovely environment, valuable debriefs! Thank you so much.”
					“Great actors, brilliantly set up, love it.”
				Educational value and relevance		Students recognised the unique educational benefit of the session, highlighting that it addressed a curricular gap and provided practical, applicable skills.	“An excellent session on something rarely covered in medical school. The simulation was a brilliant opportunity, and the debrief was very informative.”
“I think this is something that all medical students should be exposed to from early on; it was very beneficial.”							

## Discussion

This innovation improved student confidence in managing patients with LD and autism, demonstrating the value of combining knowledge-based teaching with authentic simulation.

The use of actors with lived experience enhanced authenticity and engagement. This aligns with Allport’s Contact Theory, which proposes that structured intergroup contact under conditions of equal status, cooperation, shared goals, and institutional support can reduce prejudice and improve attitudes [13]. All four conditions were met in this project.

Simulation is well established as an effective teaching tool, offering safe skill development and feedback [9-11]. Positive learner experiences are associated with stronger retention and behavioural change [14,15]. By integrating lived experience, the scenarios were not only clinically realistic but also attitudinally impactful.

The observed learning outcomes in this study align closely with Kolb’s experiential learning cycle. The simulation scenarios functioned as the ‘concrete experience’ stage, immersing students in realistic interactions with actors with lived experience of LD and autism. Quantitative improvements in confidence, particularly in assessing patients, adapting communication, and using reasonable adjustments, reflect the "reflective observation" and "abstract conceptualization" phases, during which students processed the encounter and integrated new frameworks for practice. The structured debrief facilitated by actors, carers, and faculty enabled students to reinterpret their assumptions, which is supported by qualitative feedback describing increased awareness, reduced apprehension, and greater appreciation of person-centred communication. Finally, the high post-session confidence scores and student reports that this training was "relevant," "practical," and "should be compulsory" demonstrate progression to the "active experimentation" stage, indicating readiness to apply these approaches in future clinical encounters. Together, this data shows how the intervention operationalised Kolb’s cycle and fostered meaningful cognitive and behavioural shifts consistent with experiential learning theory [12]. The lecture component, underpinned by cognitivism, efficiently transferred foundational knowledge [16]. Together, these approaches supported both knowledge acquisition and attitudinal change.

This study has several limitations. For the lecture component, pre- and post-session responses were collected anonymously and could not be matched at the individual level. As such, paired analysis was not possible, and independent sample testing (Mann-Whitney U) was used, which is less sensitive to within-person changes. The simulation subgroup was small (n = 8), limiting generalisability and statistical power; findings in this subgroup are therefore exploratory. Confidence scores relied on self-reported measures, which may not directly reflect behavioural competence or long-term retention. Participation in the simulation sessions was voluntary, introducing potential self-selection bias. Further research involving larger cohorts, matched longitudinal data, and objective skills assessment is needed to confirm and extend these findings. Broader implementation would require timetable integration and sustainable funding. Adaptations such as recorded vignettes or carer role-play may support replication in lower-resource settings. Long-term behavioural outcomes were not assessed and should be explored in future research, in line with Kirkpatrick’s framework [17].

Despite these limitations, the project addresses a critical educational gap and aligns with national priorities. This includes the NHS Long-Term Plan’s commitment to reducing health inequalities for people with LD and autism [18], GMC expectations for inclusive communication [6], and statutory obligations under the Equality Act 2010 [7].

## Conclusions

This pilot study demonstrates that a combined lecture and immersive simulation involving actors with lived experience of LD and autism is a potentially effective and feasible educational approach for improving medical students’ confidence and preparedness. The significant improvements observed in both knowledge-based and experiential outcomes suggest that this model has promise; however, conclusions are necessarily tentative given the small simulation cohort and reliance on self-reported confidence measures. The approach appears adaptable to undergraduate curricula and aligns with national priorities to improve healthcare for people with LD and autism. Further evaluation with larger samples, matched longitudinal data, and objective behavioral assessments is required to confirm effectiveness and determine sustainability before wider implementation.

## Figures and Tables

**Figure 2:**
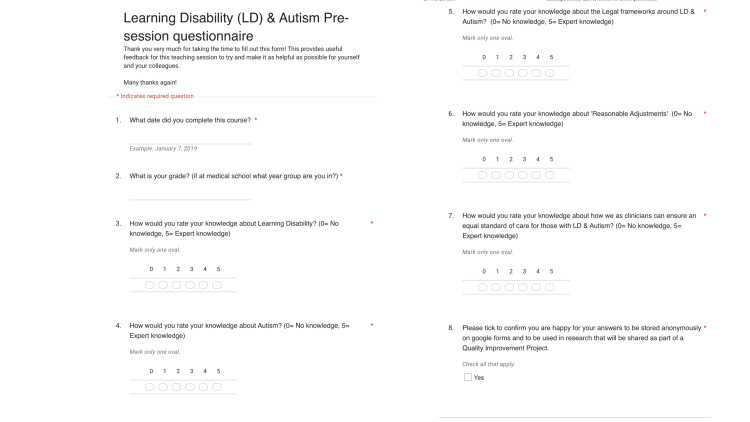
Lecture pre-session questionnaire The pre-teaching survey used to assess baseline medical student knowledge regarding key themes such as learning disability, autism, reasonable adjustments, legal frameworks and practical tools. Responses were recorded on a six-point Likert scale (0 = no knowledge to 5 = expert knowledge).

**Figure 3:**
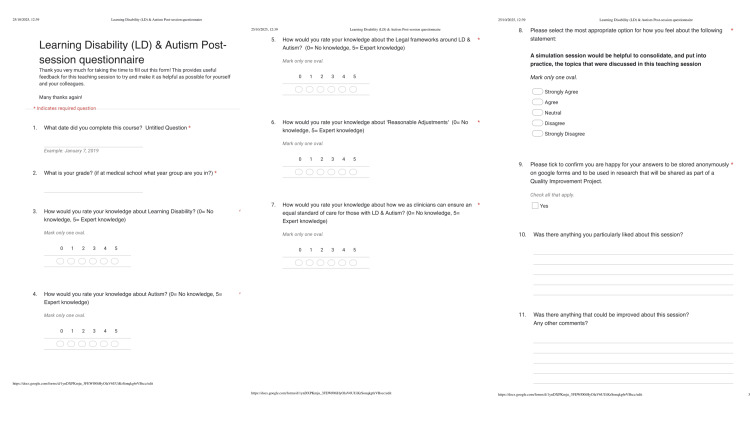
Lecture post-session questionnaire The post-lecture survey evaluating changes in medical student knowledge around key themes such as learning disability, autism, reasonable adjustments, legal frameworks, and practical tools. Responses were collected using the same six-point Likert scale as the pre-session questionnaire. Additional question for participants to rank their views on a potential simulation on the topic and to gather open-ended qualitative feedback.

**Figure 4:**
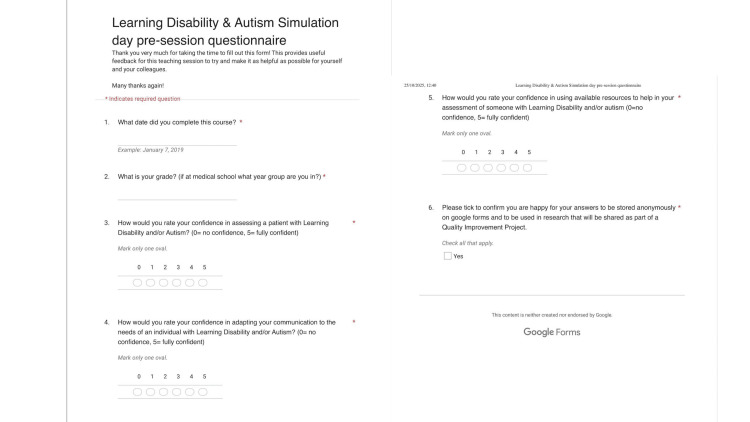
Simulation pre-session questionnaire The pre-simulation survey used to assess baseline medical student confidence around assessing, communicating and using resources when managing patients with learning disabilities and autism. Responses were collected using a six-point Likert scale (0 = no confidence to 5 = fully confident).

**Figure 5:**
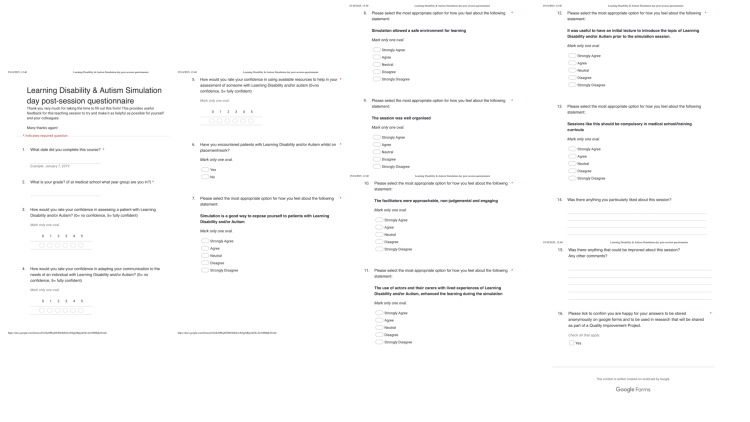
Simulation post-session questionnaire The post-simulation survey evaluating changes in medical students' confidence in assessing, communicating, and using resources when managing patients with learning disabilities and autism. Responses were collected using the same six-point Likert scale as the pre-session questionnaire. Additional questions asked to evaluate the use of simulation as a learning environment for this topic. Open-ended questions to obtain qualitative feedback also used in the form.

## Appendices

Full pre- and post-session survey instruments 
